# Correction to “Reference Intervals for Lead, Arsenic, Mercury, and Cadmium in the Population of Southwest China: A Comparative Study of Direct and Indirect Sampling Techniques”

**DOI:** 10.1002/jcla.70127

**Published:** 2025-11-10

M. Nie, H. Du, T. Xie, et al., “Reference Intervals for Lead, Arsenic, Mercury, and Cadmium in the Population of Southwest China: A Comparative Study of Direct and Indirect Sampling Techniques,” *Journal of Clinical Laboratory Analysis* 39, no. 19 (2025): e70096, https://onlinelibrary.wiley.com/doi/epdf/10.1002/jcla.70096.

In the footnote, the author's name listed as “Manging Nie” should be “Manqing Nie” and the corrected text should read as below:

Manqing Nie and Hang Du are co‐first authors, contributed equally to this study and are listed as first authors.

In Tables 3 and 4, as the 90% confidence intervals (CIs) were calculated using bootstrap resampling, the column headings currently labeled “LL (95% CI)” and “UL (95% CI)” in both tables should be updated to “LL (90% CI)” and “UL (90% CI)”, respectively.

We apologize for this error.

